# Flow mechanisms of the air-blood barrier

**DOI:** 10.1371/journal.pcbi.1012917

**Published:** 2025-04-10

**Authors:** James B. Grotberg, Francesco Romanò, John C. Grotberg

**Affiliations:** 1 Department of Biomedical Engineering, University of Michigan, Ann Arbor, Michigan, United States of America; 2 Univ. Lille, CNRS, ONERA, Arts et Métiers Institute of Technology, Centrale Lille, UMR, LMFL - Laboratoire de Mécanique des Fluides de Lille - Kampé de Fériet, Lille, France; 3 Division of Pulmonary and Critical Care Medicine, Department of Medicine, Indiana University School of Medicine, Indianapolis, Indiana, United States of America; University of Virginia, UNITED STATES OF AMERICA

## Abstract

The air-blood barrier protects the lung from blood/serum entering the air spaces, i.e., from “drowning in your own fluids”. Failure leads to pulmonary edema, a regularly fatal complication during the Covid-19 pandemic which claimed 7 million lives worldwide. Finding no mathematical models for the underlying fluid mechanics, we created the first. Governing flow equations for alveolar capillary, interstitium, and alveolus are coupled by crossflows at the capillary and epithelial membranes and end-exit flows to the lymphatics. Case examples include normal/recovery, cardiogenic pulmonary edema, acute respiratory distress syndrome, effects of positive end expiratory pressure, and a wide range of parameter values for permeability of the membranes and interstitial matrix. Previously unknown membrane fluid shear stresses calculate to values that affect cell function in many systems. We add active epithelial reabsorption which has two effects: shifting streamlines to favor alveolar-lymphatic clearance and adding to the direct alveolar-capillary clearance. Simple algebraic equations are derived for the interstitial fluid pressure, p_i_, membrane crossflow velocities and the critical capillary pressure, p_crit_, above which edema occurs. For validation, the p_crit_ predictions fit clinical definitions and flow calculations of lymphatic vs capillary clearance match animal experimental data. For decades the value of p_i_ has been imposed as an input, whereas we calculate the value as an output. They don’t agree. Since the space is too small for measurements, the ability to calculate p_i_ and p_crit_ offers new insights, questions long-held beliefs, and opens applications from physiological studies to personalized clinical care.

## Author summary

Our fluid mechanics model of the air-blood barrier provides a new conceptual framework for developing and clearing pulmonary edema. It can be used to organize data, motivate experimental investigations, interpret cause-effect relationships, understand the physical basis of disease, suggest therapies, follow interventions, and personalize care. Parameter values for the anatomy and physiology of the alveolar-capillary-interstitial layers are incorporated into fluid physics equations. We predict the alveolar interstitial pressure, which can’t be measured, uncovering several misunderstandings in pulmonary physiology and medicine. Validation is achieved by deriving an equation for the critical alveolar capillary blood pressure leading to edema and calculations of alveolar edema clearance flow rate both directly to the capillary and to the lymphatic route. Both agree with clinical and experimental measurements. The flow mechanism to lymphatics resolves a puzzle, since 1896, as to how alveolar lymphatics function from so far away, and explains the paucity of lymphatics since direct clearance to the capillary is the major route by far.

## Introduction

Pulmonary edema is broadly defined as an abnormal accumulation of fluid in lung interstitium and alveoli, and may be categorized as cardiogenic pulmonary edema (CPE) or non-cardiogenic in origin. The former is classically seen in congestive heart failure, where elevated lung capillary blood pressure drives fluid across the air-blood barrier. Approximately 1 million people in the US suffer from this condition annually [1]. Acute CPE has an in-hospital mortality rate of 30–40% [2]. The latter involves damage to that barrier leading, in severe cases, to acute respiratory distress syndrome (ARDS) [3,4]. Prior to the COVID-19 pandemic approximately 190,000 patients were diagnosed with ARDS annually in the US [5] at ~40% mortality [6]. Those numbers skyrocketed in the US, and around the world with the pandemic involving particular features related to the infection [7]. They include the acute onset of bilateral alveolar opacities, reduced lung compliance with high shunt fraction, and the classic histopathology of diffuse alveolar damage (DAD) and pulmonary vascular endothelialitis [8,9]. Pulmonary arterial catheters (PACs) used to measure pulmonary capillary wedge pressure (PCWP) ≤ 18 mmHg [10] supports the diagnosis of non-CPE.

While the focus on pulmonary edema has grown, the field has long lacked a robust mechanistic model, particularly as it pertains to alveolar microvascular fluid dynamics. Such models can be used to structure physiological studies, interpret data, sort diagnoses, promote personalization of interventions, and monitor therapeutic responses. With the experience of a worldwide COVID-19 pandemic costing nearly 7 million lives, there is compelling motivation to establish and investigate a detailed fluid mechanical model of pulmonary edema based on fundamental physics. This is quite unlike the history of fluid mechanics models for arterial blood flow, which date back to the mid-19th century. As a starting point, we recently published a model [11] as an initial step, and here add important new, clinically significant features. First, we upgrade from the Darcy porous media model to the Brinkman model. This allows us to calculate fluid flow shear stresses, especially the wall shear applied to the interstitial side of both the alveolar epithelium and the capillary endothelium. Cells are known to be biologically responsive to their shear environment. Second, we account for active fluid reabsorption that is known to occur at the alveolar epithelium. It is accomplished by imposing a constant absorption fluid velocity, v_ab_, there. Both of these new features allow further investigations, validations, and applications.

**Fig 1:**
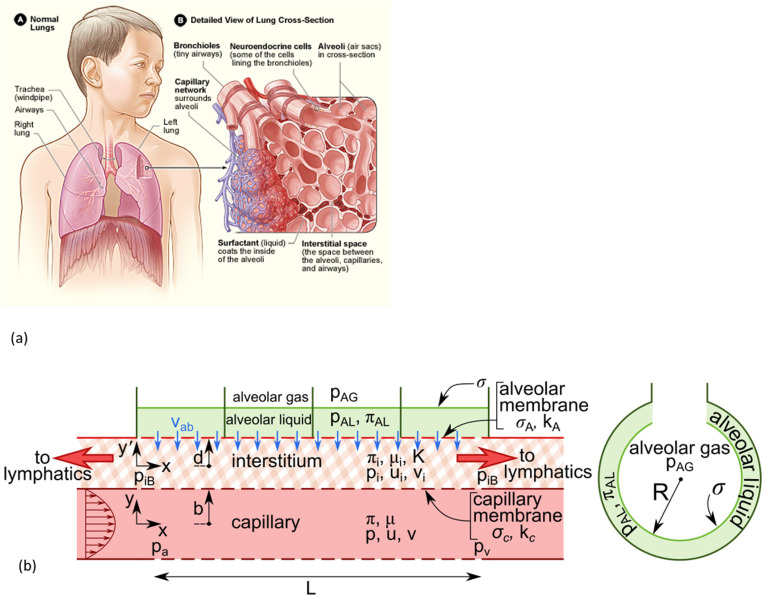
(a) Lung anatomy: (A) shows the location of the lungs and airways in the body (B) is a detailed view of the lung structures such as the bronchioles, neuroendocrine cells, alveoli, capillary network, alveolar liquid with surfactant, and interstitial space. Wikipedia, https://commons.wikimedia.org/wiki/File:Lung_structure_normal.jpg, accessed October 19, 2022. (b) A two-dimensional model of a septal tract with capillary, interstitium, and alveolar compartments. See text for definitions.

Fig 1a (A) shows the gross lung anatomy in the chest including the left and right lung and parts of the airway tree (trachea, large airways) while (B) is a small-scale view including the capillary network, alveolar liquid with surfactant, and the interstitium [12]. In a sketch of the pulmonary lobular anatomy in cross section [13], the alveoli are on the order of 100 μm in diameter and the interalveolar septa are 12 ±3 μm thick [14,15]. The capillary network runs within the septa and passes by several alveoli. Lymphatics are shown to be up to several alveoli away, i.e., hundreds of µms.

Fig 1b is our two-dimensional model of a septal tract with three compartments: capillary, interstitium and alveolus. The capillary domain is 0 ≤ x ≤ L, -b ≤ y ≤ b while the interstitium domain is 0 ≤ x ≤ L, -d ≤ y’ ≤ d. Blood flow is driven by pressure differences between the upstream arterial, p(x=0) = p_a_, and downstream venous, p(x=L) = p_v_. The blood has pressure, p, and velocity components u,v in the x,y directions, respectively. It also has osmotic pressure, π, and viscosity, μ. The interstitial fluid has pressure, p_i_, velocity components u_I_, v_i_ in the x and y’ directions, respectively. The end pressures are p_iB_ at x=0 and x=L. It also has osmotic pressure, π_i_, viscosity μ_i_, and Darcy permeability, K. The capillary membrane has hydraulic conductivity, k_c_, with reflection coefficient, σ_c_. The alveolar epithelial membrane has hydraulic conductivity, k_A_, with reflection coefficient, σ_A_, and also has an active reabsorption velocity v_ab_ [16,17]. The alveolar liquid pressure is p_AL_ and osmotic pressure is π_AL_, while the alveolar gas pressure is p_AG_. The surface tension between the alveolar gas and liquid is σ. Red arrows indicate interstitial end outflows at x=0 and x=L, which are available to the lymphatics. The lower half of the capillary and the lower interstitium strip and alveoli will be the mirror image of the upper half, so there is symmetry of the entire system with respect to the capillary centerline, y=0. An example of a spherical alveolus is shown in Fig 1b (right) with radius, R.

For prescribed values of the input parameters listed above and also in Table 1 with references, the pressure and velocity fields are calculated for the capillary, p(x,y), u(x,y), v(x,y) and for the interstitium, p_i_(x,y’), u_i_(x,y’), v_i_(x,y’).). The system is solved using Fourier series, see Tables A and B in S1 Appendix. From those solutions we characterize the underlying fluid mechanics of pulmonary edema and its clearance.

**Table 1:** Base state parameter values and references.

Dimensional Parameters			
Symbol	Base Set Value	Description	Range/Reference
b	3 μm	capillary height (2b)	6.3 [49], 8.3 [50]
d	0.4 μm	interstitium thickness (4d)	4d = 1.24 [15], 1.63 [51], 1.72 [52]
K	10^-13^ cm^2^	interstitial permeability	1.46-7.6×10^-12^ [53], 2.53×10^-14^ [54], 4.35×10^-14^ [55], 2.3×10^-11^ [56,57]
k_A_	5×10^–8^ cm·mmHg^-1^·s^-1^	alveolar membrane hydraulic conductivity	2.85×10^-7^ [58], (3.51-10)×10^-8^ [59]
k_c_	1×10^–6^ cm·mmHg^-1^·s^-1^	capillary membrane hydraulic conductivity	1×10^-7^ [60], 2-20×10^-7^ [61], 1.7×10^-7^ [62], 0.36×10^-7^ [63,64]
L	500 μm	capillary length	250-850 [65–67]
μ	0.02 poise	blood viscosity	0.025 @ 22°C, [68], reduce for 37°C
μ_i_	0.013 poise	interstitial fluid viscosity	[69]
p_a_	9 mmHg	arterial pressure	Average value 6.6 mmHg [70]
p_AG_	0 mmHg	alveolar gas pressure	
p_iB_	-7.35 mmHg	interstitial fluid end pressure	[18,19]
p_v_	6 mmHg	venous pressure	Average value 6.6 mmHg [70]
π	25 mmHg	capillary blood osmotic pressure	24.8 [71], 25.4
π_AL_	0 mmHg	alveolar liquid osmotic pressure	
π_i_	10.15 mmHg	interstitial fluid osmotic pressure	[19,32,71]
P_s_/ε=μU_s_/εb =(p_a_-p_v_)	3 mmHg	pressure scale	
ρ	1.06 g/cm^3^	blood density	
R	0.01 cm	alveolar radius	[72]
σ	4 dyn/cm	surface tension	[73]
σ_A_	0.8	alveolar reflection coefficient	[74,75]
σ_c_	0.8	capillary reflection coefficient	[74, 75]
U_s_=b^2^(p_a_-p_v_)/μL	0.36 cm/s	capillary velocity scale	
v_ab_	1×10^–3^ μm/s	alveolar absorption velocity	[43,44]
W_s_=K(p_a_-p_v_)/μ_i_d	7.7 μm/s	interstitial fluid velocity scale	

## Results

**Fig 2:**
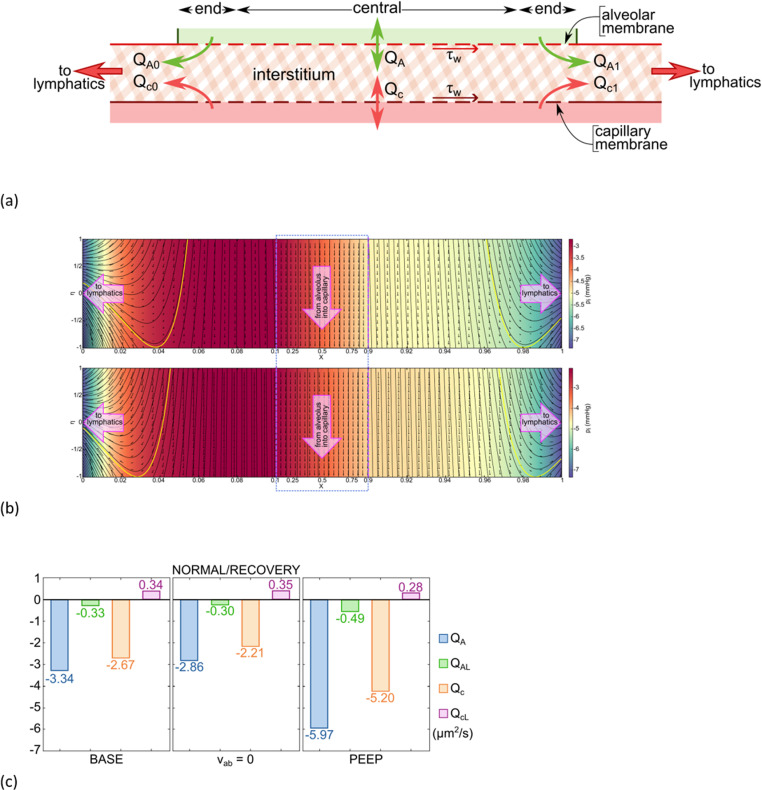
(a) Flow rate definitions across boundaries (b) Streamlines, velocity fields, and color-coded pressure field for: (b upper) the base set of parameters, BASE; (b lower) is BASE with pAG = 15 cmH2O PEEP. Note that the horizontal axis is compressed for 0.1 ≤ X ≤ 0.9 and circumscribed with a blue rectangle. The bounding streamlines in yellow separate alveolar and capillary contributions to the end-exit flows at X=0 and X=1 which become available to lymphatics., (c) Flow calculations for Normal/Recovery including BASE, PEEP and zero absorption velocity, v_ab_ = 0. Note Q_AL_ = Q_A0_ + Q_A1_ and Q_cL_ = Q_c0_ + Q_c1_. See text for details.

The definitions of calculated flows are shown in Fig 2a which is a sketch for many of our general results. Let Q_c_ be the two-dimensional volumetric flow rate across η = -1, the capillary membrane, i.e., $Q_{c}=\int_{0}^{L}v_{i}(x,\eta =-1)dx$. Let Q_A_ be the volumetric flow rate across at η = 1, the alveolar membrane, i.e., $Q_{A}=\int_{0}^{L}v_{i}(x,\eta =+1)dx$. When Q_A_ > 0 we have net alveolar edema and when Q_A_ < 0 we have net alveolar clearance. At x=0 (X=0) there are end-exit flows which are available to the lymphatics, Q_c0_ from the capillary and Q_A0_ from the alveolus. In Fig 2b those contributions are separated by a yellow bounding streamline which intersects the x=0 boundary at $\eta =\eta_{s0}$. That makes $Q_{c0}=d\int_{-1}^{\eta_{s0}}u_{i}(x=0,\eta )d\eta$ and $Q_{A0}=d\int_{\eta_{s0}}^{1}u_{i}(x=0,\eta )d\eta$. Both have negative values because u_i_ < 0 at x=0. Their sum is the two-dimensional outflow from the interstitium toward the lymphatics at x=0. At x=L (X=1), the end-exit flows consist of Q_c1_ and Q_A1_ from the capillary and alveolus, respectively, and there is a yellow bounding streamline in Fig 2b that intersects the X=1 boundary at $\eta =\eta_{s1}$. Then $Q_{c1}=d\int_{-1}^{\eta_{s1}}u_{i}(x=L,\eta )d\eta$ and $Q_{A1}=d\int_{\eta_{s1}}^{1}u_{i}(x=L,\eta )d\eta$, where both have positive values. Their sum is the two-dimensional outflow from the interstitium toward the lymphatics at x=L. Let the total contribution from the alveolus to the lymphatics be Q_AL_ = |Q_A0_| + |Q_A1_| and from the capillary to the lymphatics be Q_cL_ = |Q_c0_| + |Q_c1_|. The model prediction that lymphatic flow has two competing, simultaneous sources is not a prevalent concept in lung physiology. The system was checked for the net difference in membrane crossflows equaling the lymphatic outflows, i.e., $\left(\left|Q_{A}-\;Q_{c}\right|-\left(\left|Q_{0}\right|+\left|Q_{L}\right|\right)\right)/\left|Q_{A}-\;Q_{c}\right|=O\left(10^{-9}\right)$.

Fig 2b (upper) shows a solution to the governing equations using the input parameter values of Table 1 which we call BASE. The flow pattern is characterized with streamlines, velocity vector field, and a color-coded pressure field. The axes are X=x/L and η=y’/d. The central region, 0.1 ≤ X ≤ 0.9, has quasi one-dimensional flow in these coordinates, so is compressed in X to allow better visualization of the expanded end regions, 0.0 ≤ X ≤ 0.1 and 0.9 ≤ X ≤ 1.0, where the flow is fully two-dimensional.

In the central region, flow is from the alveolus to the capillary, i.e., clearance. In the end regions the streamlines are curvilinear and the exiting flows at X=0 and X=1 have contributions from both the capillary and the alveolus, separated by the bounding yellow streamline. These outflows become available to the lymphatics. The color-coded pressure field, p_i_(X,η), has a maximum of $p_{i}\sim -3.5\;mmHg$ and decreases gradually for increasing X along with the capillary pressure which is transmitted through the capillary Starling equation. There are relatively steep X-gradients of p_i_ in the end regions due to the proximity of the fixed end pressure, p_iB_ = -7.35 mmHg which come from subpleural measurements [18,19]. However, the central region pressure distribution has an η-dependence which is too small to reveal a color change. Nevertheless, it contributes to a dominant η-gradient of p_i_ since the denominator distance scale is very small, 2d = 0.8 μm. This accounts for the direct crossflow, v_i_, in the central region from one membrane to the other.

For Fig 2b (upper) we calculate Q_A_, Q_AL_, Q_c_, and Q_cL_, all shown in Fig 2c BASE. The percent of Q_A_ that exits the ends is$100\times \left(\left|Q_{AL}\right|\right)/\left|Q_{A}\right|$, i.e., the alveolar-lymphatic clearance. It calculates to 9.9%, while the rest, 90.1%, is the alveolar-capillary clearance percent, i.e., directly across the interstitium in the central region. We observe that the flow is bidirectional at the capillary membrane, negative in the central region, but positive in the end regions. Setting v_ab_ = 0 results in similar flow patterns to Fig 2b (upper), but shifts the bounding end-exit streamlines upward, reducing Q_AL_ from -0.33 to -0.3 μm^2^/s. This is a new effect not previously associated with active reabsorption, that v_ab_ increases alveolar-lymphatic clearance. Second, in the central region the effect of v_ab_ is along the streamlines. For v_ab_ = 0 we calculate Q_A_ = -2.86 μm^2^/s which is 0.48 μm^2^/s less than the BASE value of Q_A_ = -3.34 μm^2^/s. This is due to the reabsorption volume flow rate contribution -v_ab_ × L = -10^-3^ μm/s × 500 μm = -0.5 μm^2^/s. The absorption velocity is essentially subtracted directly in the central region, which is the traditional understanding of reabsorption.

In Fig 2b (lower) the flow calculations for BASE with p_AG_ = 15 cmH_2_O PEEP (Positive End Expiratory Pressure) are shown. PEEP is applied during acute CPE and ARDS, but continues on through recovery. The overall alveolar clearance flow is Q_A_ = -5.97 μm^2^/s, nearly double that of BASE, with -0.49 μm^2^/s exiting through the lymphatics. The bounding streamlines shift further toward the capillary compared to Fig 2b (upper) and the alveolar-lymphatic clearance percent computes to 8.2%.

**Fig 3:**
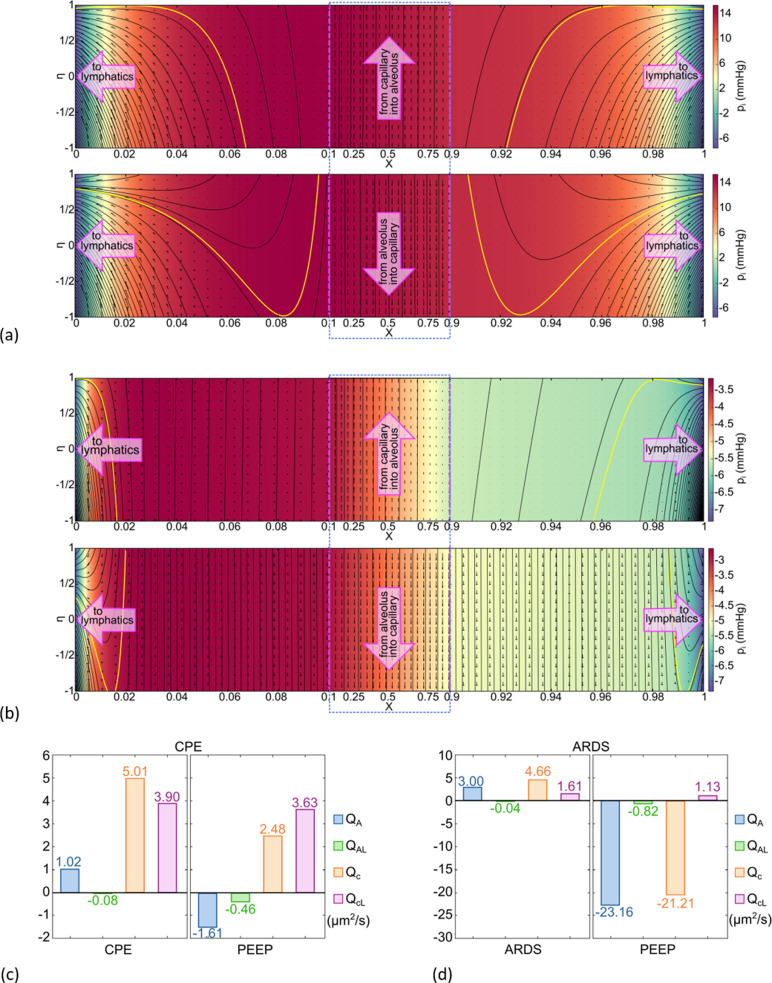
Streamlines, velocity fields, and pressure color coding for (a upper) CPE, (a lower) CPE with 15 cmH_2_O PEEP, (b upper) ARDS, (b lower) ARDS with 15 cmH_2_O PEEP. Note that the horizontal axis is compressed for 0.1 ≤ X ≤ 0.9 and circumscribed with a blue rectangle. The bounding streamlines in yellow separate alveolar and capillary contributions to the end-exit flows at X=0 and X=1 which become available to lymphatics. (c) flow calculations for (a), (d) flow calculations for (b), Note Q_AL_ = Q_A0_ + Q_A1_ and Q_cL_ = Q_c0_ + Q_c1_. See text for details.

Moving on to pulmonary edema, Fig 3a (upper) shows the results for CPE where BASE parameters are used, but with elevated blood pressure, p_a_ = 28 mmHg, p_v_ = 25 mmHg. Again, the central region, 0.1 ≤ X ≤ 0.9 is compressed. The overall flow is pulmonary edema with Q_A_ = 1.02 μm^2^/s as shown in Fig 3c. However, the flow across the capillary membrane is Q_c_ = 5.01 μm^2^/s with Q_cL_ = 3.90 μm^2^/s, so 77.9% of the cross-capillary flow exits through the ends to the lymphatics. This is a protective function that diverts capillary fluid away from the alveolus. The maximum interstitial pressure is p_i_ = +15mmHg. Again, we observe that the flow is bidirectional, but this time at the alveolar membrane, positive in the central region but negative in the end regions. Fig 3a (lower) is CPE with p_AG_ = 15 cmH_2_O PEEP and the flow is reversed to be clearance with Q_A_ = -1.61 μm^2^/s and Q_AL_ = -0.46 μm^2^/s, ~29% alveolar-lymphatic clearance. This effect of PEEP on Q_A_ is much smaller, ~ 1/4^th^, than that seen in Fig 2c for the Normal/Recovery BASE parameter set. This is primarily due to the pressures opposing PEEP clearance, p and p_i_. For Fig 2b (lower) they are in a normal range, but for Fig 3a (lower) they are significantly elevated.

Fig 3b (upper) represents results for ARDS which we model as BASE but with increased surface tension due to surfactant interference, σ = 40 dyn/cm, and increased osmotic pressure due to proteinaceous inflammation materials, π_AL_ = 10mmHg. The hydraulic conductivities are increased by a factor of ten due to enhanced leakiness from inflammation: k_A_ = 5×10^-7^ cm·mmHg^-1^·s^-1^ and k_c_ = 10^-5^ cm·mmHg^-1^·s^-1^. The central region shows flow from the capillary to the alveolus, i.e., pulmonary edema, in the face of normal capillary, p, and interstitial, p_i_, pressures. The flow rates are Q_c_ = 4.66 μm^2^/s and Q_A_ = 3.00 μm^2^/s, see Fig 3d. Fig 3b (lower) has p_AG_ = 15 cmH_2_O PEEP and changes the flows to clearance with Q_A_ = -23.2 μm^2^/s and Q_c_ = -21.2 μm^2^/s. This is a very strong effect of PEEP and reflects the 10-fold increase in both k_c_ and k_A_ which control membrane crossflow in either direction, i.e., edema or clearance. In addition, the pressures opposing clearance for ARDS, p_i_ and p, are in normal ranges, compared to PEEP used in CPE where p_i_ and p are significantly elevated. Comparing Fig 3c to Fig 2c, Q_A_ is nearly 15 times smaller with PEEP in CPE vs PEEP in ARDS, while Q_c_ has switched signs and is approximately 10 times smaller.

**Fig 4:**
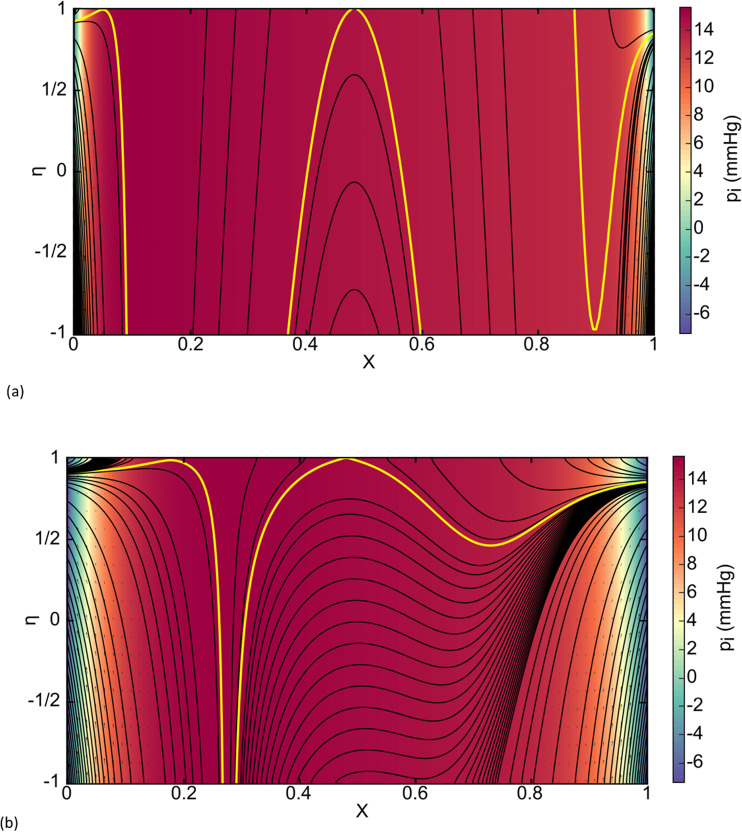
Streamlines, velocity fields, and color-coded pressure field for. (a) CPE with p_AG_ = 7 cmH_2_O PEEP, (b) CPE with 7 cmH_2_O PEEP and 10K._._

Because the capillary blood pressure is dropping in the X direction, it is possible to have edema upstream with clearance downstream. Fig 4a is CPE, but with a lower value of PEEP at p_AG_ = 7 cmH_2_O. As we have seen before, the end region bounding streamlines separate the positive capillary crossflow which exits to the lymphatics and the negative epithelial crossflow which also exit to the lymphatics. Then there is a recirculation region in the central section with a third bounding streamline. Inside that streamline flow enters and exits the interstitium which is protective. If we also then increase K by a factor of 10, labeled as 10K, Fig 4b shows the closed streamline opens downstream since X-direction velocities increase.

**Fig 5:**
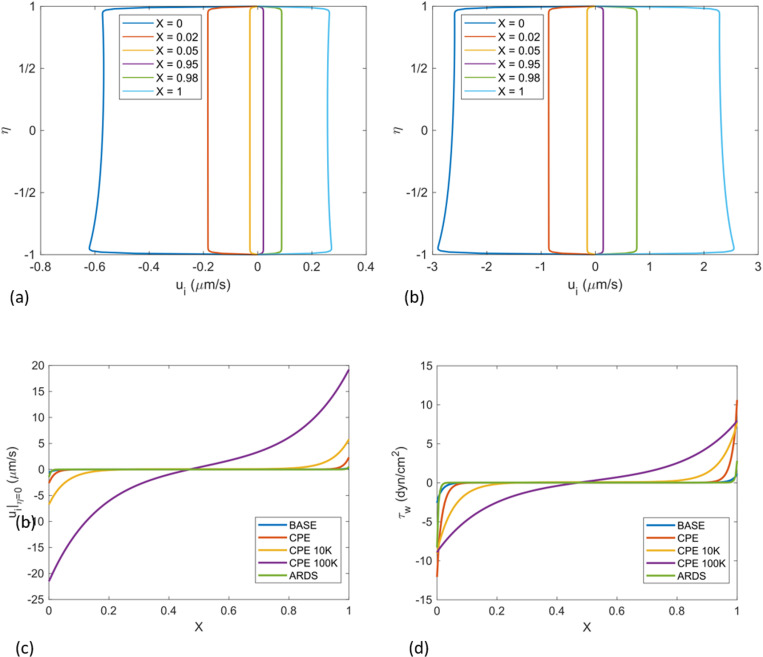
Comparison of u_i_ (η) at X=0.0, 0.02, 0.05, 0.95,0.98,1.0. for (a) BASE (b) CPE. (c) u_i_ (η=0) vs X for BASE, CPE, CPE + 10K, CPE + 100K, ARDS. (d) wall shear stress τ_w_ for the alveolar membrane for BASE, CPE, CPE with10K, CPE 100K, and ARDS.

Velocities in the X-direction, u_i_, are small in the central region but become relatively large in the end regions as the pressure X-gradients increase. Fig 5a compares the horizontal velocities u_i_(η) at X = 0.0,0.02,0.05,0.95,0.98,1.0, for BASE. The capillary pressure is highest at the arterial end, X=0, where p(X=0) = p_a_, Consequently, it drives the fastest end-exit velocity out of the interstitium there. The average u_i_ ~ -0.58 μm/s, while at the other exit, X=1, the average u_i_ ~ 0.25 μm/s. The other values of X shown in Fig 5a are very close to the respective exits. The average at X=0.5 is u_i_ = 5.8×10^-3^ μm/s which is 100 times smaller than at X=0 and similar to v_i_ at X=0.5. Note the very thin boundary layers at the alveolar and capillary membranes, η=±1, to match the no-slip conditions. This is an advantage of the Brinkman model vs the Darcy model which cannot satisfy no-slip. Fig 5b for CPE shows all of the velocities in the end regions increasing significantly by factors of ~ 5–10.

**Fig 6:**
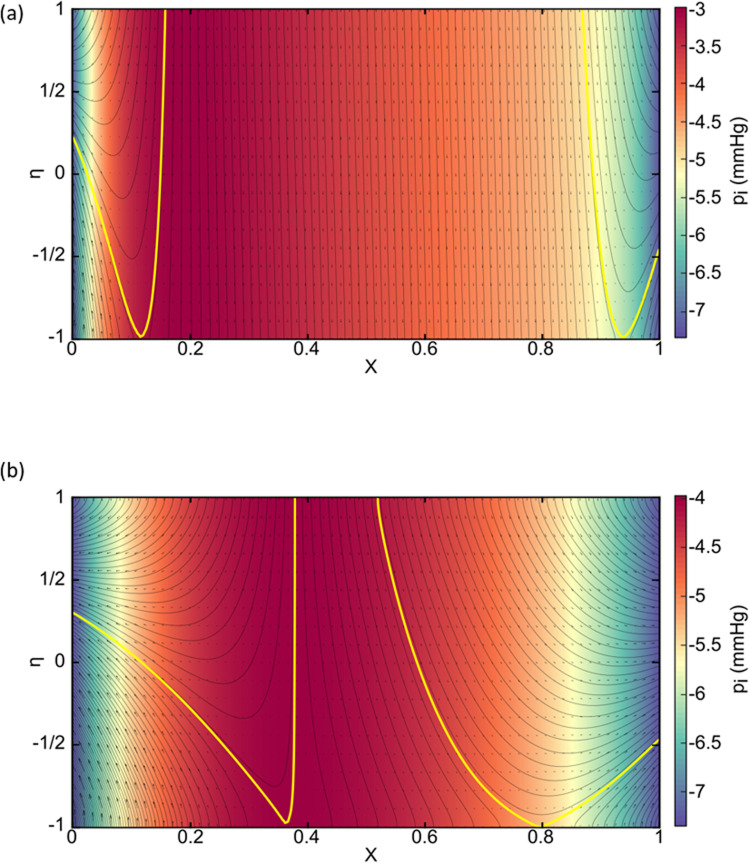
Streamlines, velocity fields, and color-coded pressure field for BASE with (a)10K and (b) 100K.

Fig 5c shows the centerline velocity u_i_ (η=0) vs X for BASE, CPE, CPE with 10K (ten times BASE K), CPE with 100K (100 times BASE K), and ARDS. Note that BASE and ARDS overlap because they have similar, normal pressure distributions, p_i_. Increasing K has a significant effect on increasing the velocities and extending the end regions while shrinking the central regions, as shown in Fig 6a and 6b for 10K and 100K. The wall shear stress on the alveolar epithelial and capillary membranes are very similar, so in Fig 5d we plot only the epithelial membrane wall shear, τ_w_ (X) corresponding to Fig 5c. The values range from τ_w_ ~ 0.02 dyn/cm^2^ in the central region to 12 dyn/cm^2^ at the ends for CPE with 100K.

## Discussion

Our model solves the pressure, velocity, and shear stress field for interstitial flow coupled to the capillary and alveolar compartments through respective Starling equations. The calculations are for an instantaneous state and do not follow physiological changes in time such as breathing motions, interstitial swelling and fluid shifts. All parameters are treated independently, so, for example, values of b, d, and K do not respond to the pressure p_i_ directly. The reabsorption of fluid across the alveolar epithelial membrane is modeled with a prescribed velocity -v_ab_ inserted into that Starling equation. We find that -v_ab_ has two effects: shifting streamlines toward the capillary in the end regions to garner more alveolar-lymphatic clearance; and, directly subtracting from crossflows in the central region to increase alveolar-capillary clearance. For the Normal/Recovery BASE state ~ 90% of edema clearance from the alveolus was directly across the interstitial layer to the capillary. The Brinkman model for the interstitium lets us calculate wall shear stresses on the membranes, and we find their range of values are known to be physiologically stimulating to both endothelial and alveolar epithelial cells in other contexts, but from the interstitial side.

With regard to the wall shear, τ_w_, calculations in Fig 4d it has been shown that fluid shear stress on alveolar type II cells in a microfluidic device can stimulate surfactant production, as studied in a range of 4–20 dyn/cm^2^ [20]. Capillary endothelial cells have been studied extensively for their response to shear stress from the blood flow [21,22]. In either case, shear stress gradients can also stimulate biological responses, which are significant in the end regions of Fig 4d. For both membranes, alveolar epithelium and capillary endothelium, our model is the first to estimate fluid shear stresses from the interstitial side.

For many applications the central region flow is essentially one-dimensional across the interstitium, with 3 resistances in series: the capillary membrane, the interstitial matrix, and the alveolar membrane. For convenience call, $p_{ic}=p_{i}(y^{\prime }=-d)$, $p_{iA}=p_{i}(y^{\prime }=d)$, $v_{i}(y^{\prime }=-d)=v_{ic}$, and $v_{i}(y^{\prime }=d)=v_{iA}$. Then the two Starling’s laws at the capillary, $v_{ic}=k_{c}\left(p-p_{ic}-s_{c}\right)$, and alveolar $v_{iA}=k_{A}\left(p_{iA}-p_{AL}-s_{A}\right)$ membranes where $s_{c}=\sigma_{c}(\pi -\pi_{i})$ and $s_{A}=\sigma_{A}\left(\pi_{i}-\pi_{AL}\right)+\left(v_{ab}/k_{A}\right)$. Using similar notation, the porous media flow across the interstitial layer is essentially Darcy since there are no tangential boundaries. Let the vertical velocity in the layer be $v_{i}=k_{i}\left(p_{ic}-p_{iA}\right)$ where $k_{i}=1333K/2\mu_{i}d$. Setting v_iA_ = v_ic_ and v_ic_ = v_i_ we can solve for p_ic_ and p_iA_. We recognize that k_i_ = 1.28×10^-4^ cm·mmHg^-1^·s^-1^ is much larger than k_A_ = 5×10^-8^ cm·mmHg^-1^·s^-1^ and k_c_ = 1×10^-6^ cm·mmHg^-1^·s^-1^_._ The resistances to flow are ordered by the size of the reciprocals, 1/k_A_>> 1/k_c_>> 1/k_i_. Clearly 1/k_A_ dominates and we can take the limit k_A_/k_i_ → 0, k_c_/k_i_ → 0 to simplify the result as


$$p_{ic}\sim p_{iA}=\frac{p-\sigma_{c}(\pi -\pi_{i})+\left(\frac{k_{A}}{k_{c}}\right)\left(\left(\frac{p_{AG}}{1.36}-\frac{2\sigma }{(1333)R}\right)+\sigma_{A}\left(\pi_{i}-\pi_{AL}\right)+\left(\frac{v_{ab}}{k_{A}}\right)\right)}{\left(1+\left(\frac{k_{A}}{k_{c}}\right)\right)}$$ (1)

The alveolar contributions are all multiplied by (k_A_/k_c_) = 0.05 for our base state, so the capillary contributions, $p-\sigma_{c}(\pi -\pi_{i})$, dominate Eq. (1). Since p decreases essentially linearly in the central region, let X=0.5 where p ~ (p_A_ + p_v_)/2. For BASE parameters Eq. (1) yields p_i_ = -3.7 mmHg (-3.2 mmHg with 15 cmH_2_O PEEP), CPE 14.4 mmHg (14.9 mmHg with 15 cmH_2_O PEEP), and ARDS -4.4 mmHg (-3.9 mmHg with 15 cmH_2_O PEEP) which equal the full solution values. For our ARDS model we multiplied both k_A_ and k_c_ by 10, so the ratio k_A_/k_c_ did not change and interstitial pressure remained normal as shown in Fig 3b.

To solve for the critical capillary blood pressure, p_crit_, which leads to edema, we substitute p_ic_ from Eq. (1) into v_ic_ and set v_ic_ = 0. The result is


$$p_{crit}=\left(\frac{p_{AG}}{1.36}-\frac{2\sigma }{(1333)R}\right)+\sigma_{c}(\pi -\pi_{i})+\sigma_{A}\left(\pi_{i}-\pi_{AL}\right)+\left(\frac{v_{ab}}{k_{A}}\right)$$ (2)

where we have substituted for p_AL_ from the Law of Laplace. Edema flow occurs when p > p_crit_, and alveolar-capillary clearance when p < p_crit_. Major clinical methods to increase p_crit_ can be seen in Eq. (2) to include increasing p_AG_, π and v_ab_ or decreasing σ and π_AL_. In many modeling approaches, the reflection coefficients are taken to be equal, σ_c_ = σ_A_, as we do, so π_i_ drops out.

Surface tension, σ, is elevated when there is a surfactant deficiency, as occurs in premature neonates [23–25] or ARDS where surfactant function is hampered. These neonates develop pulmonary edema which is reversed with surfactant therapy, indicating that their alveolar capillary blood pressure is above p_crit_ with high surface tension and then below it after treatment [26]. One of the major pulmonary targets of COVID-19 are the alveolar type II cells [27] reducing production. The effect of reabsorption velocity, v_ab_, is to increase p_crit_ as one might expect. It appears as v_ab_/k_A_ = 2 mmHg in BASE. It is reassuring that our parameter choices from the wide literature lead to this reasonable result for reabsorption. Interestingly, k_A_ tends to increase in ARDS, as membranes become leakier due to inflammation, thereby reducing the protective effect of v_ab_, just when it is needed.

For our base state parameter set, BASE, Eq. (2) yields p_crit_ = 21.4mmHg,19.4 mmHg for vab=0. These values are consistent with the clinical setting where (PCWP) > 18 mmHg is considered diagnostic for CPE [10]. This is why our CPE examples led to pulmonary edema. The value drops to p_crit_ = 6.2 mmHg for ARDS causing edema for normal vascular pressures. Adding PEEP of p_AG_ = 15 cmH_2_O to ARDS increased p_crit_ = 17.2 mmHg, allowing clearance. PEEP of p_AG_ = 15 cmH_2_O applied to CPE led to clearance in the central region and contributions to the end-exit flows from both capillary and alveolus. However, PEEP of p_AG_ = 7 cmH_2_O applied to that CPE led to upstream edema but downstream clearance. These comparisons constitute model validation for its one-dimensional predictions. Validation of two-dimensional predictions hinges on measurements of lymphatic outflow from the lung. Here are three examples.

**Fig 7:**
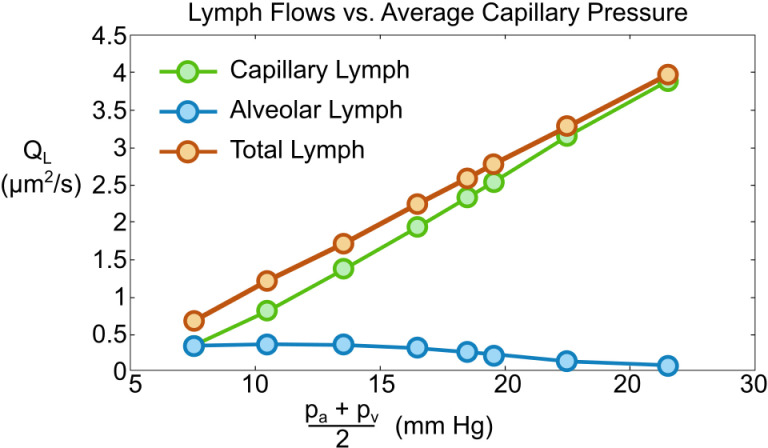
Lymph flow vs average capillary pressure (p_a_ +pv)/2.

In [28] left atrial pressure (LAP) was increased stepwise in unanesthetized sheep, while measuring the lung efferent lymph duct output. They found lymphatic flow increased approximately linearly with LAP. We have calculated our total lymphatic output, (Q_AL_ + Q_cL_) vs the average capillary pressure, (p_a_ + p_v_)/2, for the pressure pairs (p_a_,p_v_) = (9,6), (12,9), (15,12), (18,15), (20,17), (21,18), (24,21), (28,25) mmHg. The results show an excellent linear curve fit (Q_AL_ + Q_cL_) = 0.18(p_a_ + p_v_)/2 - 0.64, R² = 0.99, see Fig 7. They [28] also determined that lymph protein concentration decreased approximately linearly with LAP from sieving of capillary fluid. We found linearly increasing Q_cL_ = 0.190(p_a_ + p_v_)/2 - 1.16, R² = 0.99 but decreasing Q_AL_ = -0.016(p_a_ + p_v_)/2 + 0.52, R² = 0.84. An increasing share of the end-exit flow comes from the capillary, which dilutes the protein level in the lymphatic fluid from washout.

Sheep studies following acute hydrostatic pulmonary edema [29] were studied for overall clearance, determined from decreasing extravascular lung water (EVLW), while measuring the lymphatic contribution. Lymphatics contribute between 8.8 to 14.6% of total clearance. Using our BASE model we found the alveolar-lymphatic clearance percent was 9.9% while total lymphatic flow was $100\times \left(\left|Q_{AL}\right|+\;\left|Q_{cL}\right|\right)/\left|Q_{A}\right|\sim 20\%$. This percentage reduces to 12.5% by increasing L to 800µm since |Q_A_| increases from the longer surface while $\left|Q_{AL}\right|+\;\left|Q_{cL}\right|$ remains essentially the same. Other investigators have found alveolar-lymph clearance is 4% [30] and 18% [31] of the total as discussed in [32].

In dog experiments [33], σ was increased by treatment with inhaled aerosol of dioctyl sodium sulfosuccinate. LAPs were step-wise increased to cause edema. Controls with normal σ showed lymphatic flow increases over baseline for elevated LAP. However, treated dogs exhibited much larger lymphatic flow rates for similar LAP, since the pressures were further above the lowered. For example, increasing the surface tension by a factor of 10, σ = 40 dyn/cm, leads to p_crit_ = 16 mmHg, so more lung units are susceptible to edema. Our model fits these three sets of experimental results providing two-dimensional validation.

Previous experimental studies of the general topic [34–37] involve pressure measurements in subpleural interstitium to include interalveolar junctions and perivascular adventitia of 50μm venules. This is a significantly different mechanical environment from what we are studying: alveolar septal fluid mechanics. A more global approach [34] involves pressures measured by micropuncture both in the subpleural alveolar interstitium and the hilum. The investigators find a pressure difference tending to cause flow to the hilum, a proposed mechanism of clearance from the alveoli. However, the intervening alveolar septa have pressures, p_i_, which we calculate to be higher than both the hilum and subpleural pressures, so any flow from the subpleura to the hilum would need to circumnavigate that pressure barrier.

Traditional lung physiology [38] describes the alveolar fluid balance through the capillary Starling equation alone, with p = 7mmHg, p_i_ = -8 mmHg, π = -28 mmHg, π_I_ = -14 mmHg and σ_A_ = σ_c_ = 1. The constant value p_i_ is an input, taken from subpleural measurements similar to our p_iB_ of -7.35 mmHg [18,19]. It does not agree with our calculated output values in the central region which are more positive and not constant. The sum [38] is +1 mmHg causing flow across the capillary membrane into the interstitium. It is presented that lymphatics in the interstitium pump out the excess fluid, but may not keep up leading to edema. This explanation of pulmonary edema forms the teaching basis to 10 million physicians and 20 million nurses world-wide. In reality, however, lymphatics are up to hundreds of μm away from alveoli [13], i.e., too far to suck interstitial fluid over that distance. The mechanism presented in our model, that the capillary pressure is transmitted to the interstitium over a finite membrane length, drives interstitial fluid toward the lymphatics. It resolves a puzzle since 1896 [11,39] as to how alveolar lymphatics function from so far away. The model also can explain the relative paucity of lymphatics in the alveoli, since alveolar-capillary clearance carries a majority of the load compared to alveolar-lymphatic clearance.

The model not only provides a novel understanding of pulmonary interstitial clearance in both normal and diseased states, but also provides a simple equation, Eq. (2), with variables that can be readily manipulated by the clinician at the bedside to improve clearance of the pulmonary interstitium. For example, pulmonary venous pressure, p_v_, in both CPE and non-CPE may be reduced with diuretics while capillary oncotic pressure, π, may be increased with intravenous albumin in proteinemic patients. Glucocorticoids reduce elevated k_c_, k_A_ in select patients with ARDS. Notably, the application of PEEP can be individualized in both disease states to improve the direction of fluid-flow from alveoli to capillary. In the wake of the COVID-19 pandemic, there have been numerous publications devoted to the personalization of PEEP in ARDS to optimize alveolar recruitment and limit alveolar overdistension using bedside tools such as esophageal manometry (P_es_) and electrical impedance tomography (EIT) [40,41]. The application and titration of PEEP is often thought of as a method for alveolar recruitment to improve tidal volume distribution, lung mechanics, and pulmonary gas exchange. In “non-recruitable” patients, PEEP may over-distend open alveoli resulting in volutrauma and ventilator-induced lung injury. However, PEEP is not currently perceived as a method to improve clearance of alveolar and interstitial edema. The above model would allow the bedside clinician to calculate the PEEP required to lower p_crit_ and improve interstitial edema clearance, which could play an integral role in PEEP-personalization in both CPE and non-CPE alike. Further studies and animal models are needed to integrate this novel model into clinical applications.

## Methods

Capillary blood flow is modeled using lubrication theory leading to a locally parabolic profile. In our previous model [11] we used Darcy’s law for the interstitium. However, here we use the Brinkman model, $\left(\mu_{i}/K\right){\underset{―}{u}}_{i}=-\underset{―}{\nabla }p_{i}+\mu_{i}\nabla^{2}{\underset{―}{u}}_{i}$ which allows us to calculate shear stresses. Conservation of mass for the interstitium yields Laplace’s equation for p_i_, $\nabla^{2}p_{i}=0$. The end pressures are set to a constant p_i_(x=0)=p_i_(x=L)=p_iB_. Crossflow at the permeable capillary membrane is given by the Starling equation [42] $v(y=b)=k_{c}\left(p-p_{i}(y^{\prime }=-d)-\sigma_{c}(\pi -\pi_{i})\right)$. We also need to match the crossflow velocities there, $v_{i}(y^{\prime }=-d)=v(y=b)$ The alveolar membrane boundary condition is also modelled using a Starling equation, however this membrane has active transport processes for water and salt [16,17] which can help to resolve pulmonary edema over time. We propose a unique modification by subtracting an absorption velocity term, v_ab_ > 0, as follows $v_{i}=k_{A}\left(\left(p_{i}-p_{AL}\right)-\sigma_{A}\left(\pi_{i}-\pi_{AL}\right)\right)-v_{ab}$.

We can estimate v_ab_ from rat data on the overall rate of volume absorption [43] given in as 0.49±0.02 ml/h under normal conditions and can be ~ 40% higher for Congestive Heart Failure. Measurements of rat alveolar surface area [44] are in the range of 2,000 cm^2^ at an inflation of 24% TLC. Dividing the flow rate by the surface area yields $v_{ab}\sim \;7x10^{-4}\mu m/s$. This value can be an underestimate, since not all of the available alveolar surface area likely participates in reabsorption. However, it can also be an overestimate, since passive processes from the hydraulic and osmotic pressures also lead to clearance to capillary and lymphatics. We will choose v_ab_ = 10^-3^ μm/s. There are a number of clinical situations which will reduce v_ab_ [45] including hypoxia [46], ARDS [47], and congestive heart failure [48].

We use the following base parameter values (BASE): b = 3 μm, d = 0.4 μm, K = 10^-13^ cm^2^, L = 500 μm, μ = 0.02 poise, μ_i_ = 0.013 poise, p_a_ = 9 mmHg, p_v_ = 6 mmHg, p_AG_ = 0 mmHg, σ = 4dyn/cm, p_iB_ = -7.35 mmHg, π = 25 mmHg, π_AL_ = 0 mmHg, π_I_ = 10.15 mmHg, k_A_ = 5x10^-8^ cm·mmHg^-1^·s^-1^, k_c_ = 1x10^-6^ cm·mmHg^-1^·s^-1^, v_ab_ = 10^-3^ μm/s, R=0.01 cm, σ_A_ = σ_c_ = 0.8. Detailed discussion of these choices from extensive references are found in [11] and Table 1. Using the Law of Laplace, we calculate the alveolar liquid pressure, p_AL_ in mmHg from the alveolar gas pressure, p_AG_, in cmH_2_O and the surface tension, σ, in dyn/cm, as $p_{AL}=p_{AG}/1.36-2\sigma /(1333)R$ using the conversion factors 1 mmHg = 1.36 cmH_2_O and 1 mmHg = 1333 dyn/cm^2^.

## Supporting information

S1 Appendix Detailed solution method. Table A. Dimensionless parameters. Table B. Dimensional and dimensionless variables.
(DOCX)
