# Supplementary material for: Flow mechanisms of the air-blood barrier
Source: PLoS Comput Biol. 2025 Apr 10;21(4):e1012917. doi: 10.1371/journal.pcbi.1012917 (PMC12052194; doi:10.1371/journal.pcbi.1012917)
Supplement: S1 Appendix — Table A. Dimensionless parameters. Table B. Dimensional and dimensionless variables. (DOCX) [file pcbi.1012917.s001.docx]

S1 Appendix

Capillary blood flow

Momentum conservation in the 2D capillary is governed by the Navier-Stokes equation simplified for lubrication theory. In dimensionless form the X and Y components are

$$-\frac{\partial P}{\partial X}+\frac{\partial^{2}U}{\partial Y^{2}}=0 , \frac{\partial P}{\partial Y}=0$$

where U and V are the X and Y velocity components and P is the pressure. The coordinate value ranges are $0\leq X\leq1 , -1\leq Y\leq1$. Eq. assumes $\varepsilon=b/L<<1$ and$\varepsilon\mathrm{Re}<<1$ where Re is the capillary Reynolds number which, itself, satisfies $\mathrm{Re}<<1$. From the Y-component of Eq. we learn that P is independent of Y, so $P=P(X)$. Integrating the X-component of Eq. leads to the solution form of U,

$$U=-\frac{1}{2}\frac{dP}{dX}\left(1-Y^{2}\right)$$

where $U(Y=\pm1)$ satisfies no-slip at the capillary membranes and we see that U is locally parabolic.

From conservation of mass, $\partial U/\partial X+\partial V/\partial Y=0$, the Y-component of velocity, V, is

$$V=-\int_{0}^{Y} \frac{\partial U}{\partial X} dY=\frac{1}{2}\frac{d^{2}P}{dX^{2}}\left(Y-\frac{Y^{3}}{3}\right)$$

which satisfies the symmetry condition V(Y=0) = 0.

Interstitium

In our previous model (11) we used Darcy’s law for the interstitium modelled as a porous media. Here we conserve momentum using the Brinkman equation, $\left(\mu_{i}/K\right)\underline{u}_{i}=-\underline{\nabla}p_{i}+\mu_{i}\nabla^{2}\underline{u}_{i}$, where the pressure gradient is balanced both by a Darcy resistance contribution and also a Navier-Stokes viscous contribution. In dimensionless form it is

$$\underline{U}_{i}=-\underline{\nabla} P_{i}+Da\nabla^{2}\underline{U}_{i}$$

where $U_{i} , V_{i}$are the dimensionless velocities in the $\xi, \eta$ directions, respectively, and $P_{i}$is the dimensionless pressure. The dimensionless coordinate value ranges are $0\leq\xi\leq\lambda, -1\leq\eta\leq1$. Note that $Da=K/d^{2}$is the Darcy number and Da = 0 yields Darcy’s law. An advantage of the Brinkman equation is that it allows us to calculate wall shear stresses on the capillary and epithelial cell membranes, see τ_w_ in Figure 2(a) ,

Conservation of mass for the interstitium is $\underline{\nabla}\cdot\underline{U}_{i}=0$ which, after inserting Eq. , yields Laplace’s equation for $P_{i}$

$$\nabla^{2}P_{i}=0$$

Membranes

The crossflow at the permeable capillary membrane is given by the Starling equation (42) in dimensional form $v(y=b)=k_{c}\left(p-p_{i}(y'=-d)-\sigma_{c}(\pi-\pi_{i})\right)$. Using the dimensionless variables, substituting for $V(Y=1)$from Eq. , Starling’s equation becomes a differential equation for P coupled to $P_{i}$ in the $\xi$ variable

$$\frac{d^{2}P}{d\xi^{2}}-\kappa_{c}^{2}P=\kappa_{c}^{2}\left(-P_{i }(\eta=-1)-S_{c}\right)$$

We also need to match the crossflow velocities at the endothelial boundary, $v_{i}(y'=-d)=v(y=b)$. In dimensionless form this condition is

$$V_{i }(\eta=-1)=\beta V(Y=1)$$

The permeable alveolar membrane boundary condition is also modelled using Starling’s equation, however this membrane has active transport processes for water and salt (16, 17) which can help to resolve pulmonary edema over time. We propose to modify the Starling equation at the alveolar epithelium by including an absorption velocity term, v_ab_ > 0 , as follows $v_{i}=k_{A}\left(\left(p_{i}-p_{AL}\right)-\sigma_{A}\left(\pi_{i}-\pi_{AL}\right)\right)-v_{ab}$ where the negative sign indicates flow from the alveolus to the interstitium, i.e. clearance. We can estimate v_ab_ from rat data on the overall rate of volume absorption (43) given in as $0.49\pm0.02\text{ }ml/h$under normal conditions and can be ~ 40% higher for Congestive Heart Failure. Measurements of rat alveolar surface area (44) are in the range of 2,000 cm^2^ at an inflation of 24% TLC. Dividing the flow rate by the surface area yields $v_{ab}\sim\text{ }7x10^{-4}\mu m/s$. This value can be an underestimate, since not all of the available alveolar surface area likely participates in reabsorption. However, it can also be an overestimate, since passive processes from the hydraulic and osmotic pressures also lead to clearance to capillary and lymphatics, There are a number of clinical situations which will reduce v_ab_ (45) including hypoxia (46), ARDS (47), and congestive heart failure (48). In dimensionless form the flux across the alveolar membrane is

$$V_{i}\left(\eta=1\right)=\kappa_{A}^{2}\left(\left(P_{i}\left(\eta=1\right)-P_{AL}\right)-S_{A}\right)-V_{ab}$$

Using the Law of Laplace we calculate the alveolar liquid pressure, p_AL_ in mmHg from the alveolar gas pressure, p_AG_, in cmH_2_O and the surface tension, σ, in dyn/cm, as

$$p_{AL}=\frac{p_{AG}}{1.36}-\frac{2\sigma}{\left(1333\right) R}$$

using the conversion factors 1 mmHg = 1.36 cmH_2_O and 1 mmHg = 1333 dyn/cm^2^.

Solution

As in (11), we solve the above system of equations and boundary conditions using Fourier analysis. For interstitial end boundary pressures, $P_{i }=P_{iB}$at $\xi=0,\lambda$, let the leading order pressure field be given by the Fourier sine series

$$P_{i}=P_{iB}+\sum_{n=1}^{N} f_{n}(\eta)\sin\left(k_{n}\xi\right)$$

where $k_{n}=n\pi/\lambda$ where N=2,000 in our computations. Inserting Eq. into Eq. yields a differential equation for $f_{n}(\eta)$

$$f_{n}^{' '}-k_{n}^{2}f_{n}=0$$

whose general solution is $f_{n}=a_{n}e^{k_{n}\eta}+b_{n}e^{- k_{n}\eta}$. Using the capillary membrane flux, Eq. , and substituting for $P_{i }(\eta=-1)$from Eq. yields

$$\frac{d^{2}P}{d\xi^{2}}-\kappa_{c}^{2} P=-\kappa_{c}^{2}\left(P_{iB}+\sum_{n=1}^{\infty} \left(a_{n}e^{- k_{n}}+b_{n}e^{k_{n}}\right)\sin\left(k_{n}\xi\right)\right)-\kappa_{c}^{2}S_{c}$$

Substituting $\xi=\lambda X$, the solution to Eq. , now in terms of X, is given by

$$P=\left(c_{0}e^{\kappa_{c}\lambda X}+d_{0}e^{-\kappa_{c}\lambda X}\right)+S_{c}+P_{i B}+\sum_{n=1}^{\infty} c_{n}\sin\left(n\pi X\right)$$

where the first bracketed term is the homogeneous solution, involving the coefficients $c_{0} , d_{0}$, and the rest is the particular solution.

By inserting Eq. into Eq. the values of c_n_ are found, and then imposing the upstream and downstream blood pressure conditions on Eq. , $P(X=0)=P_{a} , P(X=1)=P_{v}$, determine c_0_ and d_0_. The results are

$$\begin{matrix} c_{n}=\kappa_{c}^{2}\left(a_{n}e^{- k_{n}}+b_{n}e^{k_{n}}\right)/\left(k_{n}^{2}+\kappa_{c}^{2}\right) \\ c_{0}=\left(\left(P_{iB}+S_{c}-P_{a}\right)\text{e}^{-\kappa_{c}\lambda}+\left(-P_{iB}-S_{c}+P_{v}\right)\right)/\left(\text{e}^{\kappa_{c}\lambda}-\text{e}^{-\kappa_{c}\lambda}\right) \\ d_{0}=-\left(\left(P_{iB}+S_{c}-P_{a}\right)\text{e}^{\kappa_{c}\lambda}+\left(-P_{iB}-S_{c}+P_{v}\right)\right)/\left(\text{e}^{\kappa_{c}\lambda}-\text{e}^{-\kappa_{c}\lambda}\right) \end{matrix}$$

The solutions for U, V, come from substituting for P, from Eq. , into Eq. and Eq.

$$\begin{matrix} U=-\frac{1}{2}\left(1-Y^{2}\right)\left(\kappa_{c}\lambda c_{0}e^{\kappa_{c}\lambda X}-\kappa_{c}\lambda d_{0}e^{-\kappa_{c}\lambda X}+\sum_{n=1}^{\infty} n\pi c_{n}\cos\left(n\pi X\right)\right) \\ V=\frac{1}{2}\left(Y-\frac{Y^{3}}{3}\right)\left(\left(\kappa_{c}\lambda\right)^{2}c_{0}e^{\kappa_{c}\lambda X}+\left(\kappa_{c}\lambda\right)^{2}d_{0}e^{-\kappa_{c}\lambda X}-\sum_{n=1}^{\infty} n^{2}\pi^{2}c_{n}\sin\left(n\pi X\right)\right) \end{matrix}$$

For the interstitial velocities let U_i_ be the cosine series

$$U_{i}=\sum_{n=1}^{N} A_{n}(\eta)\cos\left(k_{n}\xi\right)$$

and insert into Eq. . Collecting terms leads to an differential equation for A_n_(η)

$$Da A_{n}^{' '} - \left(1+Da k_{n}^{2}\right)A_{n}=k_{n}\left(a_{n}e^{k_{n}\eta}+b_{n}e^{- k_{n}\eta}\right)$$

The solution for A_n_  is

$$A_{n}=\left(B_{n}e^{q_{n}\eta}+C_{n}e^{- q_{n}\eta}\right)+\left(D_{n}e^{k_{n}\eta}+E_{n}e^{- k_{n}\eta}\right)$$

where $q_{n}=\left(Da^{-1}+k_{n}^{2}\right)^{1/2}$, $D_{n}=-k_{n}a_{n}$, and $E_{n}=-k_{n}b_{n}$. The first bracketed term is the homogeneous solution while the second term is the particular solution. Now enforce no-slip conditions on the membranes at $\eta=\pm1$, $A_{n}(1)=A_{n}(-1)=0$

$$\begin{matrix} A_{n}(1)=\left(B_{n}e^{q_{n}}+C_{n}e^{- q_{n}}\right)+\left(D_{n}e^{k_{n}}+E_{n}e^{- k_{n}}\right) =0 \\ A_{n}(-1)=\left(B_{n}e^{-q_{n}}+C_{n}e^{q_{n}}\right)+\left(D_{n}e^{-k_{n}}+E_{n}e^{ k_{n}}\right)=0 \end{matrix}$$

From Eq. we can solve for B_n_ and C_n_ in terms of a_n_ and b_n_.

Solving for V_i_ from conservation of mass yields

$$V_{i}=-\int\frac{\partial U_{i}}{\partial\xi} d\eta=\sum_{n=1}^{N} \left(\frac{k_{n}}{q_{n}}\left(B_{n}e^{q_{n}\eta}-C_{n}e^{- q_{n}\eta}\right)+\left(D_{n}e^{k_{n}\eta}-E_{n}e^{- k_{n}\eta}\right)\right)\sin\left(k_{n}\xi\right)$$

Then matching flow between the blood and interstitium at the capillary membrane, Eq. ,

$$\begin{matrix} \sum_{n=1}^{N} \left(\frac{k_{n}}{q_{n}}\left(B_{n}e^{-q_{n}}-C_{n}e^{ q_{n}}\right)+\left(D_{n}e^{-k_{n}}-E_{n}e^{ k_{n}}\right)\right)\sin\left(n\pi X\right)= \\ \frac{\beta}{3}\left(\left(\kappa_{c}\lambda\right)^{2}c_{0}e^{\kappa_{c}\lambda X}+\left(\kappa_{c}\lambda\right)^{2}d_{0}e^{-\kappa_{c}\lambda X}-\sum_{n=1}^{\infty} n^{2}\pi^{2}c_{n}\sin\left(n\pi X\right)\right) \end{matrix}$$

Further manipulation is needed to express the exponentials in their own sine series:

$$\begin{matrix} e^{\kappa_{c}\xi}=\sum_{n=1}^{\infty} g_{n}\sin\left(k_{n}\xi\right) , g_{n}=\frac{2n\pi\left(1-e^{\kappa_{c}\lambda}\left(-1\right)^{n}\right)}{\left(\pi^{2}n^{2}+\kappa_{c}^{2}\lambda^{2}\right)} \\ e^{-\kappa_{c}\xi}=\sum_{n=1}^{\infty} h_{n}\sin\left(k_{n}\xi\right) , h_{n}=\frac{2n\pi\left(1-\left(-1\right)^{n}\text{e}^{-\kappa_{c}\lambda}\right)}{\left(\pi^{2}n^{2}+\kappa_{c}^{2}\lambda^{2}\right)} \end{matrix}$$

Substitute Eq. into Eq. and collect terms leads to an equation in terms of a_n_ and b_n_ .

$$\left(\frac{k_{n}}{q_{n}}\left(B_{n}e^{-q_{n}}-C_{n}e^{ q_{n}}\right)+\left(D_{n}e^{-k_{n}}-E_{n}e^{ k_{n}}\right)\right)=\frac{\beta}{3}\left(\left(\kappa_{c}\lambda\right)^{2}c_{0}g_{n}+\left(\kappa_{c}\lambda\right)^{2}d_{0}h_{n}-n^{2}\pi^{2}c_{n}\right)$$

Inserting Eq. and Eq. into Eq. for the alveolar epithelial membrane gives us

$$\begin{matrix} \sum_{n=1}^{N} \left(\frac{k_{n}}{q_{n}}\left(B_{n}e^{q_{n}}-C_{n}e^{- q_{n}}\right)+\left(D_{n}e^{k_{n}}-E_{n}e^{- k_{n}}\right)\right)\sin\left(n\pi X\right) \\ =\kappa_{A}^{2}\left(\left(P_{iB}+\sum_{n=1}^{N} f_{n}(1)\sin\left(n\pi X\right))-P_{AL}\right)-S_{A}\right)-V_{ab} \end{matrix}$$

Again, we need to express all terms in the form of sine series. Let

$$\kappa_{A}^{2}\left(P_{iB}-P_{AL}-S_{A}\right) -V_{ab}=\sum_{n=1}^{\infty} d_{n}\sin\left(k_{n}\xi\right) , d_{n}=\frac{-2\left(\left(\kappa_{A}^{2}\left(P_{iB}-P_{AL}-S_{A}\right)-V_{ab}\right)\left(\left(-1\right)^{n}-1\right)\right)}{n\pi}$$

Equating coefficients, we find a second equation in terms of a_n_ and b_n_ ,

$$\left(\frac{k_{n}}{q_{n}}\left(B_{n}e^{q_{n}}-C_{n}e^{- q_{n}}\right)+\left(D_{n}e^{k_{n}}-E_{n}e^{- k_{n}}\right)\right)=\kappa_{A}^{2}\left(a_{n}e^{k_{n}}+b_{n}e^{-k_{n}}\right)+d_{n}$$

The two equations, Eq. and Eq., are solved for the remaining two unknown coefficients, $a_{n} , b_{n}$.

| Dimensionless Parameters | |
| --- | --- |
| Symbol | Value |
| β = ε^2^γdb/K | 2.81 |
| D = d/b | 0.1333 |
| Da = K/d^2^ | 6.25×10^-5^ |
| ε = b/L | 0.006 |
| γ = μ_i_/μ | 0.65 |
| k_n_ = nπ/λ |  |
| κ_A_ = (k_A_μ_i_ d/K)^1/2^(1/1333)^1/2^ | 0.014 |
| κ_c_ = (3k_C_μD^2^/b)^1/2^(1/1333)^1/2^ | 0.0000516 |
| λ = L/d = 1/εD | 1250 |
| P_a_ = p_a_/(p_a_-p_v_) | 3 |
| P_AL_ = p_AL_/(p_a_-p_v_) | -0.2 |
| P_iB_ = p_iB_/(p_a_-p_v_) | -2.45 |
| P_v_ = p_v_/(p_a_-p_v_) | 2 |
| Re = ρbU_s_/μ | 0.0057 |
| S**_A_** = σ_A_(π_i_-π_AL_)/(p_a_-p_v_) | 2.71 |
| S**_c_** = σ_c_(π-π_i_)/(p_a_-p_v_) | 3.96 |
| V_ab_ = v_ab_/W_s_ | 1.3×10^-4^ |

Table A. Dimensionless parameters.

| Dimensional Variables | |
| --- | --- |
| Symbol | Description |
| p | capillary blood pressure |
| p_i_ | Interstitial fluid pressure |
| u | capillary blood x-velocity |
| u_i_ | interstitial fluid x-velocity |
| v | capillary blood y-velocity |
| v_i_ | interstitial fluid y'-velocity |
| x | horizontal coordinate |
| y | capillary vertical coordinate |
| y' | interstitial vertical coordinate |
| Dimensionless Variables | |
| P=p/(p_a_-p_v_) | capillary blood pressure |
| P_i_=p_i_/(p_a_-p_v_) | Interstitial fluid pressure |
| U=u/U_s_ | capillary blood X-velocity |
| U_i_ =u_i_/W_s_ | interstitial fluid X-velocity |
| V=v/εU_s_ | capillary blood Y-velocity |
| V_i_ =v_i_/W_s_ | interstitial fluid η-velocity |
| X=x/L | capillary horizontal coordinate |
| ξ=x/d | interstitial horizontal coordinate |
| Y=y/b | capillary vertical coordinate |
| η=y'/d | interstitial vertical coordinate |

Table B. Dimensional and dimensionless variables.
